# Ultrasound-assisted enzymatic extraction improves the antiinflammatory activity of red dragon fruit peel pectin

**DOI:** 10.55730/1300-0152.2797

**Published:** 2026-02-05

**Authors:** Bao LE, Seung Hwan YANG, Hoang Dang Khoa TA

**Affiliations:** 1 Research Group in Pharmaceutical and Biomedical Sciences, Faculty of Pharmacy, Ton Duc Thang University, Ho Chi Minh City, Vietnam; 2 Department of Biotechnology, Chonnam National University, Yeosu, Republic of Korea; 3 School of Computer Science, Duy Tan University, Da Nang, Vietnam; 4 DTU AI and Data Science Hub, Duy Tan University, Da Nang, Vietnam

**Keywords:** Antiinflammatory activity, degree of esterification, galacturonic acid, red dragon fruit peel, structural characterization

## Abstract

**Background/aim:**

Inflammatory bowel disease is a chronic inflammatory disorder characterized by excessive immune activation, primarily mediated by nitric oxide and proinflammatory cytokines. This condition encompasses ulcerative colitis and Crohn’s disease, both of which significantly impair patients’ quality of life and pose persistent challenges in clinical management. Pectin, a complex carbohydrate found in plant cell walls, has shown promise in reducing inflammation.

**Materials and methods:**

In this study, pectin was extracted from red dragon fruit peel using two methods: ultrasound-assisted extraction and ultrasound-assisted enzymatic extraction. The structural characteristics and antiinflammatory effects of the extracted pectin were evaluated in lipopolysaccharide-stimulated RAW 264.7 macrophages.

**Results:**

Ultrasound-assisted enzymatic extraction produced pectin with a higher yield (19.38%), a lower degree of esterification (46.51%), a reduced molecular weight (94.67 kDa), and a greater galacturonic acid content (67.51%) compared with ultrasound extraction alone. Both pectin preparations were noncytotoxic to RAW 264.7 macrophages and significantly reduced nitric oxide production and the expression of key inflammatory mediators. Structural analysis confirmed the integrity of the pectin molecules.

**Conclusion:**

These findings suggest that ultrasound-assisted enzymatic extraction enhances the physicochemical properties and antiinflammatory activity of pectin, thereby supporting its potential application in future studies targeting inflammatory bowel disease.

## 1. Introduction

Inflammatory bowel disease (IBD), encompassing ulcerative colitis and Crohn’s disease, has emerged as a major chronic disorder worldwide (Ng et al., 2017). Although current therapies—such as biological agents, antibiotics, immunosuppressants, and antiinflammatory drugs—may be effective, they are frequently associated with unpredictable adverse effects (Pithadia and Jain, 2011; Appiah et al., 2025). Inflammation is a fundamental defense mechanism mediated by nitric oxide (NO) and proinflammatory cytokines; however, when dysregulated or excessive, it contributes to the development of chronic conditions, including asthma, rheumatoid arthritis, atherosclerosis, cancer, and IBD (Libby, 2007). Recent studies have explored the therapeutic potential of plant-derived bioactive compounds in modulating immune responses and alleviating inflammation. For example, grape seed proanthocyanidin extract has been shown to attenuate arthritis in a murine model by modulating T-cell subsets and suppressing key inflammatory mediators, suggesting that natural products may regulate both proinflammatory and antiinflammatory pathways to facilitate disease amelioration (Ahmad et al., 2013). In light of these challenges, increasing attention has been directed toward the development of safe and effective therapeutic strategies that not only alleviate inflammation but also promote intestinal barrier integrity.

Pectin, in particular, has shown promise as a bioactive polysaccharide derived from food sources, exhibiting antiinflammatory effects both in vitro and in vivo (Beukema et al., 2020; Donadio et al., 2024). It represents a promising adjunct or alternative to conventional pharmacological therapies, with the potential to mitigate inflammation while supporting overall health and reducing adverse effects.

Pectin is a structural heteropolysaccharide located in the primary cell walls of most plants and is primarily composed of a linear polymer of (1→4)-linked α-D-galacturonic acid, referred to as homogalacturonan, which may be partially methyl-esterified at the C-6 position. Pectin is classified according to its degree of esterification (DE) into high-methoxyl pectin (HMP; DE > 50%) and low-methoxyl pectin (LMP; DE < 50%) (Mohnen, 2008). LMP has been reported to exhibit greater bioactivity than HMP (Hu et al., 2020; Picot-Allain et al., 2022). Commercially, pectin is typically extracted from fruit pomace or peel using strong acids under prolonged high-temperature conditions, processes that are time- and energy-intensive and may result in polymer degradation (Srivastava and Malviya, 2011). Recent systematic reviews and human intervention studies have demonstrated that pectin supplementation may effectively reduce serum cholesterol levels in individuals with mild hypercholesterolemia and improve gastrointestinal outcomes in patients receiving enteral nutrition in intensive care settings (Weber et al., 2025). Furthermore, clinical trials have indicated that citrus low-methoxyl pectin may reduce inflammatory markers and anxiety in healthy volunteers, whereas modified citrus pectin has demonstrated potential therapeutic benefits in patients with advanced solid tumors (Azémar et al., 2007; Vijay et al., 2024).

Ultrasound-assisted enzymatic extraction (UAE) has gained increasing attention as an efficient technique for enhancing pectin recovery by reducing energy consumption and extraction time while improving yield and physicochemical quality. Previous studies have demonstrated that UAE can produce pectin with desirable physicochemical properties, including lower molecular weight (Mw), higher galacturonic acid content, a lower degree of esterification, and enhanced solubility compared with conventional acid extraction methods (Bosch and Malgas, 2023). The selection of extraction parameters—such as enzyme type, enzyme concentration, ultrasound power, and extraction duration—is critical for optimizing both the yield and the functional properties of the extracted pectin. For example, Qian et al. (2018) reported that the maximum pectin yield from dragon fruit peel was achieved under optimal extraction conditions, including a cellulase activity of 104 U, an ultrasonication power of 105 W, and a solution-to-sample ratio of 8.5 mL/g. Similarly, Nguyen and Pirak (2019) demonstrated that ultrasound-assisted extraction of polysaccharides from white dragon fruit peel was most effective at incubation temperatures ranging from 45 °C to 75 °C, extraction times between 30 and 60 min, and a constant solid-to-liquid ratio of 1:30 (w/v).

Several studies have demonstrated the role of pectin in the attenuation of inflammatory responses. Wu et al. (2021) used lipopolysaccharide (LPS)-activated RAW 264.7 macrophages to evaluate the antiinflammatory activity of low-molecular-weight pectin, demonstrating significant inhibition of LPS-stimulated tumor necrosis factor alpha (TNF-α), interleukin-6 (IL-6), and interleukin-1 beta (IL-1β) secretion. Similarly, citrus pectin with a degree of esterification of 90% significantly inhibited the expression of inducible nitric oxide synthase (iNOS), cyclooxygenase-2 (COX-2), TNF-α, and IL-6 via the NF-κB and AP-1 signaling pathways (Chen et al., 2006).

Currently, relatively few studies have systematically investigated the extraction conditions, structural characterization, and biological activities of red dragon fruit polysaccharides using ultrasound-assisted extraction, high-pressure thermal extraction, and alkaline extraction methods (Qian et al., 2022; Zhang and Cai, 2023). Despite these findings, the role of pectin derived from red dragon fruit peel in modulating LPS-stimulated macrophage inflammatory signaling remains insufficiently explored. Therefore, this present study was designed to examine the effects of pectin extracted from red dragon fruit peel (RDFP) using ultrasound-assisted extraction with or without enzymatic assistance (hereafter referred to as RDFP-U and RDFP-UAE, respectively) on inflammatory responses in LPS-stimulated macrophages. The antiinflammatory properties of the extracted pectin samples were evaluated through a comprehensive series of experiments, including analyses of physicochemical characteristics, NO production, cytokine secretion, and proinflammatory gene expression. These findings provide a scientific basis for the potential application of RDFP-derived pectin in the food and pharmaceutical industries.

## 2. Materials and methods

### 2.1. Materials

Red dragon fruits were harvested in Tien Giang, Vietnam. The peels were then removed, washed, and cut into 2 cm pieces. Prior to extraction, the peels were dried in a hot-air oven at 60 °C until a constant weight was reached, subsequently ground into fine RDFP powder using a pulverizer, and passed through a 60-mesh sieve.

### 2.2. Ultrasound extraction of RDFP

The dried RDFP powder was extracted in deionized water under the following conditions: a solid-to-liquid ratio of 1:30 (w/v), a temperature of 55 °C, an ultrasonic power of 120 W, and an extraction time of 75 min. Extraction was performed using an ultrasonic bath (USC0340D; Infitek Inc., Shandong, China) with internal dimensions of 24.0 cm × 13.5 cm × 10.0 cm, a capacity of 3.2 L, and a frequency of 40 kHz. After extraction, the supernatant was collected by filtration through gauze, followed by centrifugation at 1700 × *g* for 15 min. The resulting solution was concentrated to one-quarter of its original volume using a rotary evaporator. The concentrate was then deproteinized using the Sevag method, precipitated with 80% ethanol, and lyophilized to obtain crude RDFP-U. The final product was stored in silica gel desiccators.

The polysaccharide yield was calculated using the following formula:


(1) $$\text{Yield}\;(\%)={\text{m}}_{1}\;(\text{g})/{\text{m}}_{0}(\text{g})\times 100$$

where m_1_ represents the weight of the extracted polysaccharides (g) and m_0_ represents the initial weight of the dried RDFP powder (g).

### 2.3. Ultrasound-assisted enzymatic extraction of RDFP

The dried RDFP powder was extracted in 100 mL of deionized water at a solid-to-liquid ratio of 1:30 (w/v). The optimized extraction conditions included an extraction time of 75 min, a pH of 5.0, and a temperature of 55 °C. A complex enzyme mixture (cellulase:pectinase:papain, 1:1:1) was added at a total dosage of approximately 300 mg. After enzymatic extraction, the reaction mixture was incubated in a water bath at 90 °C for 10 min to inactivate the enzymes. After extraction, the supernatant was collected by filtration through gauze, followed by centrifugation at 1700 × *g* for 15 min. The resulting solution was concentrated to one-quarter of its original volume using a rotary evaporator. The concentrate was then deproteinized using the Sevag method, precipitated with 80% ethanol, and lyophilized to obtain crude RDFP-UAE. The yield of RDFP-UAE was calculated using Equation (1).

### 2.4. Purification of RDFPs

The purification of RDFP-U and RDFP-UAE was performed using a DEAE-52 cellulose ion-exchange chromatography column. Elution was performed initially with deionized water, followed by stepwise elution using NaCl solutions at concentrations of 0.2, 0.4, 0.6, 0.8, and 1.0 mol/L. The eluted RDFP-U and RDFP-UAE fractions were concentrated by rotary evaporation, dialyzed against distilled water, and subsequently freeze-dried. Further purification was conducted using a Sephadex G-200 gel filtration chromatography column (Cytiva Life Sciences, Vienna, Austria), eluted with ultra-pure water at a flow rate of 0.5 mL/min. Fractions of 3 mL were collected per tube, yielding a total of 50 tubes. Total carbohydrate content was determined using the phenol–sulfuric acid method. Based on the elution profile, the target fractions were pooled, dialyzed, and freeze-dried to obtain purified RDFP-U and RDFP-UAE.

### 2.5. Structural characterization of RDFPs

The molecular weight distribution of RDFP-U and RDFP-UAE was determined using high-performance gel permeation chromatography, as described by Le et al. (2022), with slight modifications. Briefly, the samples were eluted with 0.2 mol/L NaCl at a flow rate of 0.5 mL/min, and the column temperature was maintained at 45 °C. The copper-binding method described by Voragen et al. (1986) was used to determine the DE of RDFP-U and RDFP-UAE. The monosaccharide composition of RDFP-U and RDFP-UAE was analyzed using high-performance liquid chromatography (HPLC), as described by Le et al. (2021), with slight modifications. The RDFP samples were completely hydrolyzed with 0.5 mL of 4 M trifluoroacetic acid at 121 °C for 2 h, followed by derivatization with 1-phenyl-3-methyl-5-pyrazolone in methanol and subsequent neutralization with 0.1 mol/L HCl. The aqueous phase was filtered and subsequently subjected to HPLC analysis. Fourier transform infrared (FT-IR) spectra of RDFP samples were recorded using a VERTEX 70v FT-IR spectrometer (Bruker, Ettlingen, Germany) over a scanning range of 4000–400 cm^−1^.

### 2.6. Cell culture and RDFP treatment

The RAW 264.7 murine macrophage cell line was obtained from CLS Cell Lines Service GmbH (400319; Köln, Germany). The cells were maintained in Dulbecco’s Modified Eagle Medium supplemented with 10% fetal bovine serum and 1% penicillin–streptomycin and incubated at 37 °C in a humidified atmosphere containing 5% CO_2_.

The effects of RDFP-U and RDFP-UAE on the viability of RAW 264.7 macrophages were assessed using the MTT assay. Briefly, log-phase cells were seeded into 96-well plates at a density of 2 × 10^4^ cells per well and cultured for 24 h. Then, 100 μL of RDFP-U and RDFP-UAE at varying concentrations (0–800 μg/mL) was added to the cells and incubated for an additional 24 h. Following this incubation, the supernatants were discarded, and MTT solution was added to each well, followed by a 4 h incubation at 37 °C. After incubation, the medium was removed, and 100 μL of dimethyl sulfoxide was added to each well to dissolve the purple formazan crystals. Subsequently, absorbance was measured at 570 nm to assess cell viability.

### 2.7. Griess reaction and ELISA assays

To investigate the potential antiinflammatory properties of RDFP-U and RDFP-UAE, nitrite levels in the cell culture supernatant collected after 24 h of LPS stimulation were measured using a Griess reagent kit (G7921; Invitrogen, Waltham, MA, USA). The levels of cytokines, including IL-6, interleukin-10 (IL-10), and TNF-α, were measured using mouse ELISA kits (Elabscience Biotechnology Co. Ltd., Wuhan, China), whereas the prostaglandin E_2_ (PGE_2_) concentration in the supernatant was determined using an ELISA kit (Enzo Life Sciences, Switzerland) according to the manufacturer’s instructions. ELISA values were normalized to the total protein concentration measured using the Bradford assay.

### 2.8. Biochemical markers

The expression levels of iNOS, COX-2, and proinflammatory cytokines, including TNF-α, IL-6, and IL-1β, were evaluated based on previously described methods (Le et al., 2024), with minor modifications. Briefly, total RNA from RAW 264.7 macrophages was isolated and quantified using a SpectraMax QuickDrop spectrophotometer (Molecular Devices LLC, San Jose, CA, USA). After cDNA synthesis, RT-qPCR was performed using PanGreen Universal SYBR Green Master Mix (Bio-Helix Co. Ltd., Keelung, Taiwan), with an initial denaturation at 95 °C for 1 min, followed by 40 cycles of denaturation at 95 °C for 15 s and annealing/extension at 60 °C for 40 s. The expression levels of the target genes were normalized to glyceraldehyde-3-phosphate dehydrogenase as the internal reference gene and quantified using the 2*^-^*^ΔΔCt^ method.

### 2.9. Statistical analysis

Statistical analysis was performed using GraphPad Prism software (version 9.0; GraphPad Software, LLC, San Diego, CA, USA). One-way analysis of variance (ANOVA) followed by Tukey’s post hoc test was used to evaluate differences among groups. Differences among groups (n = 3 independent experiments) were considered statistically significant at p < 0.05.

## 3. Results and Discussion

The primary objective of this study was to assess the antiinflammatory potential of pectin extracted from red dragon fruit peel using ultrasound-assisted extraction (RDFP-U) and ultrasound-assisted enzymatic extraction (RDFP-UAE) in LPS-stimulated RAW 264.7 macrophages. The findings indicate that both RDFP-U and RDFP-UAE were noncytotoxic and significantly reduced key inflammatory markers, including nitric oxide production, iNOS and COX-2 expression, PGE_2_ synthesis, and the secretion of proinflammatory cytokines such as IL-6, IL-1β, and TNF-α, which are implicated in the pathogenesis of rheumatoid arthritis (RA) (Meroni and Valesini, 2014). Notably, RDFP-UAE demonstrated greater inhibitory effects than RDFP-U, which may be associated with its enhanced physicochemical properties. These results highlight the potential of RDFP-derived pectin, particularly when obtained via ultrasound-assisted enzymatic extraction, as a functional ingredient for further investigation in inflammation-related disorders.

### 3.1. Effect of extraction methods on RDFPs

**Figure 1:**
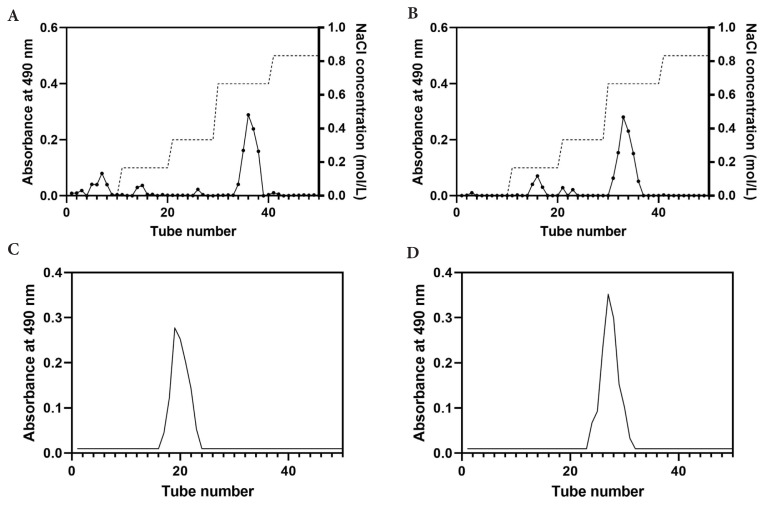
Elution profiles of RDFP-U and RDFP-UAE extracted from red dragon fruit during isolation and purification using DEAE-52 cellulose anion-exchange chromatography (A,B) and Sephadex G-200 gel filtration chromatography (C,D).

The crude polysaccharides (RDFP-U and RDFP-UAE) were isolated using DEAE-52 cellulose anion-exchange chromatography columns. The elution curve was plotted with the number of tubes as the x-axis and absorbance as the y-axis (Figures 1A and 1B). The main elution peaks were clearly symmetrical and corresponded to tubes 34–38 and 31–36, respectively. The solutions in the tubes corresponding to these main peaks were collected and further purified by Sephadex G-200 gel filtration chromatography (Figures 1C and 1D). The single, symmetrical elution peaks observed for both polysaccharides indicated effective purification. The solutions from the peak tubes were collected, dialyzed, freeze-dried, and stored at −20 °C for later use.

Based on the comparison of RDFP-U and RDFP-UAE in Table, ultrasound-assisted enzymatic extraction of RDFP resulted in a higher yield than ultrasound extraction alone, with a maximum yield of 19.38%. Therefore, UAE may be considered a more effective method for RDFP extraction under the conditions evaluated in this study. These findings are consistent with previous reports indicating that ultrasound-assisted enzymatic extraction offers high extraction efficiency (Chen et al., 2020; Wang et al., 2024). However, several extraction strategies for RDFP remain insufficiently characterized, and future studies may explore the combined application of different techniques to optimize extraction efficiency and product quality.

As shown in Table, the degree of esterification (DE) of RDFP-UAE was lower than that of RDFP-U (46.51% versus 75.82%). It is proposed that ultrasound may disrupt interactions between polysaccharides and proteins, thereby promoting their separation. Additionally, ultrasound treatment has been reported to disrupt cell wall structures, induce cleavage of pectin side chains and methoxyl groups, and reduce the molecular weight of polysaccharides (Marić et al., 2018; Córdova et al., 2022). These effects may enhance polysaccharide solubilization, thereby increasing polysaccharide content while reducing protein impurities (Cumpstey, 2013).

The degree of esterification determines the physicochemical properties of extracted pectins. Typically, pectins are classified into two categories: high-methoxyl pectin (HMP; DE > 50%) and low-methoxyl pectin (LMP; DE < 50%). Based on its DE value, RDFP-UAE can be categorized as LMP.

In general, Mw significantly influences the bioactivity and potential applications of extracted pectins. Previous studies using different extraction methods reported RDFP molecular weights ranging from 88 to 298 kDa (Muhammad et al., 2014; Qian et al., 2018). As shown in Table, the Mw of RDFP-UAE (94.67 kDa) was lower than that of RDFP-U (178.25 kDa). This result suggests that ultrasound-assisted enzymatic extraction resulted in greater polysaccharide depolymerization than ultrasound extraction alone under the conditions evaluated. Chen et al. (2022) reported that sonication combined with natural deep eutectic solvents reduced polysaccharide molecular chain size as ultrasound intensity increased, regardless of solvent type. Similarly, Shang et al. (2019) reported that the Mw of *Medicago sativa* L. polysaccharides extracted by UAE was 13,013.1 kDa lower than that obtained by enzymatic extraction and 2620.3 kDa lower than that obtained by ultrasound extraction alone. These findings are consistent with previous reports indicating that ultrasound-induced cavitation may cleave glycosidic linkages in pectin polysaccharides, thereby producing lower-Mw fractions (Wang et al., 2024). Therefore, UAE may be considered a suitable approach for obtaining RDFP with lower molecular weight under the experimental conditions evaluated.

The compositional characteristics of RDFP extracted with and without enzymatic assistance are presented in Table. Galacturonic acid was the predominant monosaccharide in both samples, with a significantly higher content observed in the UAE-extracted samples (67.51%). This observation is consistent with the findings of Abou-Elseoud et al. (2021), who reported that sonication significantly increased galacturonic acid content in sugar beet pectin. Additionally, monosaccharides derived from nonpectic polysaccharides (e.g., glucose and mannose) were considerably lower in pectin obtained after combined ultrasound and enzyme treatment, consistent with other UAE studies (Ma et al., 2016; Marić et al., 2018). These results suggest that UAE may enhance pectin purity compared to ultrasound extraction alone. Generally, galacturonic acid content is considered an indicator of pectin purity, whereas the presence of other neutral sugars may reflect residual nonpectic polysaccharides.

**Figure 2:**
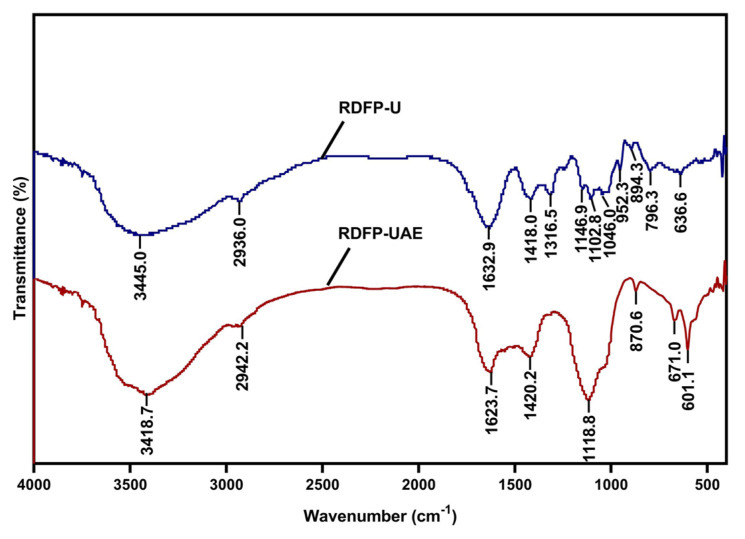
FT-IR spectra of RDFP-U and RDFP-UAE extracted from red dragon fruit.

Furthermore, FT-IR analysis was conducted to examine the effects of different extraction methods on the functional groups of RDFP. Figure 2 shows the spectral profiles of RDFP extracted with and without enzymatic assistance. The broad absorption bands at 3445.0 cm^−1^ and 3418.7 cm^−1^ correspond to O–H stretching vibrations associated with intermolecular and intramolecular hydrogen bonding in galacturonic acid chains (Wang et al., 2019). The bands at 2936.0 cm^−1^ and 2942.2 cm^−1^, assigned to C–H stretching vibrations (−CH, −CH_2_, −CH_3_), were similar across samples, suggesting that no major alterations occurred in the main polysaccharide backbone during sonication (Li et al., 2017). Peaks near 1729 cm^−1^ were attributed to esterified carboxyl groups (–COOR), whereas peaks at 1418.0 cm^−1^ and 1420.2 cm^−1^ were associated with aliphatic C–H deformation vibrations (Guo et al., 2019). Additional peaks at 1316, 1146, 1102, and 1046 cm^−1^ corresponded to C–OH stretching vibrations and C–O–C glycosidic bond vibrations, reflecting glycosidic linkages between monosaccharide units. Differences between RDFP-U and RDFP-UAE were mainly observed in the fingerprint region (1100–600 cm^−1^), which reflects monosaccharide composition and glycosidic bonding patterns. Overall, FT-IR analysis indicated that both samples exhibited characteristic polysaccharide functional groups, with spectral differences consistent with variations in chemical composition and Mw.

### 3.2. Effects of RDFPs on cell viability, NO production, and iNOS expression in RAW 264.7 cells

**Figure 3:**
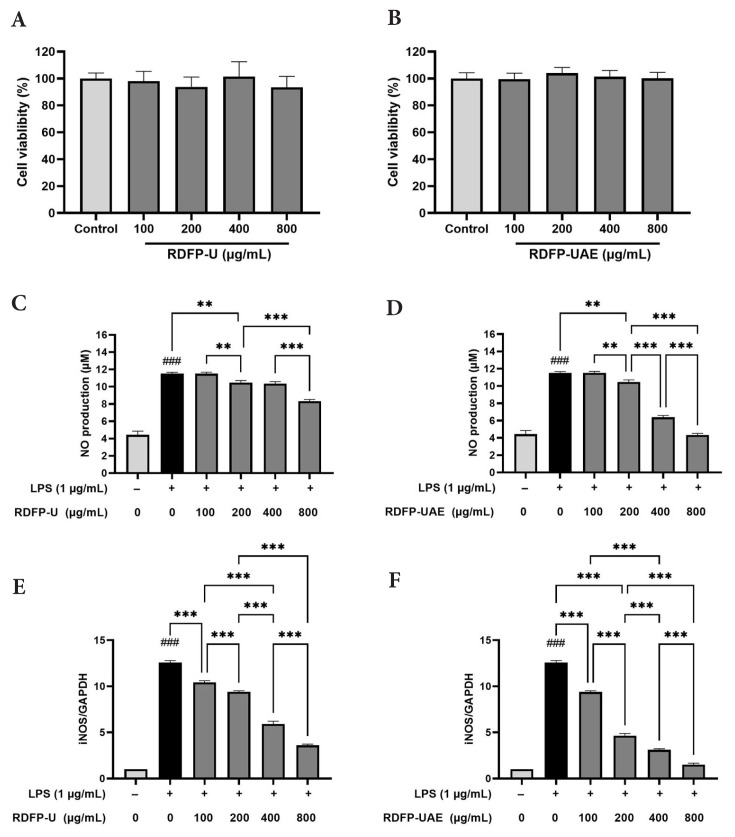
Effects of RDFP-U and RDFP-UAE extracted from red dragon fruit on cell viability and NO production in RAW 264.7 cells. (A,B) Cell viability of RAW 264.7 cells treated with different concentrations (100, 200, 400, and 800 μg/mL) of RDFP-U and RDFP-UAE for 24 h. (C,D) Nitric oxide (NO) production in RAW 264.7 cells. (E,F) mRNA expression of iNOS in RAW 264.7 cells pretreated with RDFP-U and RDFP-UAE at different concentrations (100, 200, 400, and 800 μg/mL) for 4 h, followed by cotreatment with lipopolysaccharide (LPS, 1 μg/mL) for an additional 12 h. Data are presented as mean ± standard error of the mean (SEM) (n = 3). p < 0.001 versus control; ** p < 0.01; *** p < 0.001 versus LPS.

The MTT assay was performed to evaluate the effects of RDFP-U and RDFP-UAE on RAW 264.7 cell viability. The results showed that neither RDFP-U nor RDFP-UAE had a significant impact on the viability of RAW 264.7 cells (Figures 3A and 3B). This finding is consistent with previous reports indicating that pectin exhibits minimal cytotoxicity toward RAW 264.7 macrophages and may promote cell proliferation under certain conditions (Nattha et al., 2021; Zhu et al., 2021). Therefore, subsequent antiinflammatory experiments were conducted using safe concentrations of 100–800 μg/mL in RAW 264.7 cells treated with 1 μg/mL LPS.

In antiinflammatory studies, nitric oxide (NO) production in macrophages typically occurs in response to cytokine stimulation or microbial challenge. To assess the potential antiinflammatory properties of RDFP-U and RDFP-UAE, NO levels were measured in cell culture supernatants. NO production significantly increased following LPS stimulation (1 μg/mL for 12 h), whereas treatment with RDFP-U and RDFP-UAE suppressed NO production in LPS-stimulated cells in a dose-dependent manner (Figures 3C and 3D). At 800 μg/mL, RDFP-U reduced NO production by 27.67%, whereas RDFP-UAE reduced NO production by 62.38%, suggesting greater inhibitory activity for RDFP-UAE.

The expression of iNOS at the mRNA level showed a significant increase following LPS stimulation. Treatment with RDFP-U and RDFP-UAE at concentrations of 100, 200, 400, and 800 μg/mL resulted in a dose-dependent reduction in iNOS expression in LPS-stimulated cells (Figures 3E and 3F). At 800 μg/mL, RDFP-UAE reduced relative iNOS expression to 1.507, compared with 3.607 for RDFP-U, indicating a stronger inhibitory effect of RDFP-UAE under these conditions.

### 3.3. Effects of RDFPs on PGE_2_ production and COX–2 expression

**Figure 4:**
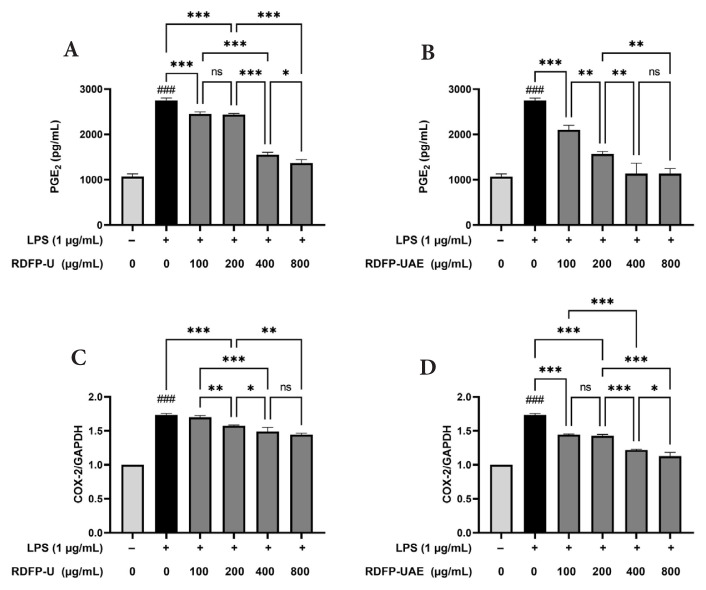
Effects of RDFP-U and RDFP-UAE extracted from red dragon fruit on (A) PGE_2_ production and (B) COX-2 expression in RAW 264.7 cells treated with different concentrations (100, 200, 400, and 800 μg/mL) for 24 h. Data are presented as mean ± SEM (n = 3). ns, not significant; ### p < 0.001 versus control; * p < 0.05; ** p < 0.01; *** p < 0.001 versus LPS.

Next, the inhibitory effects of RDFP-U and RDFP-UAE on PGE_2_ production and COX-2 expression were evaluated in LPS-stimulated RAW 264.7 cells. Both PGE_2_ production and COX-2 mRNA expression showed a statistically significant increase (p < 0.001) following LPS stimulation (Figure 4). Treatment of RAW 264.7 cells with RDFP-U and RDFP-UAE at concentrations of 100, 200, 400, and 800 μg/mL resulted in a significant, concentration-dependent decrease in LPS-induced PGE_2_ production. Treatment with 100 μg/mL RDFP-UAE significantly inhibited LPS-stimulated COX-2 mRNA expression, whereas RDFP-U showed no significant effect. Additionally, RDFP-UAE at 800 μg/mL markedly suppressed COX-2 mRNA levels in LPS-stimulated cells, suggesting that the reduction in PGE_2_ production may be associated with decreased COX-2 expression in activated macrophages.

COX-2 is an inducible enzyme that converts arachidonic acid into prostaglandin E_2_ (PGE_2_), a key mediator of inflammation (Jin et al., 2023). Under inflammatory stimuli such as LPS, COX-2 expression increases, elevating PGE_2_ levels and amplifying macrophage-driven inflammatory responses. In this study, RDFP-UAE treatment significantly suppressed COX-2 mRNA expression and reduced PGE_2_ production in LPS-stimulated RAW 264.7 cells in a dose-dependent manner. These findings suggest that RDFP-UAE exerts antiinflammatory effects that may involve the downregulation of COX-2 expression and related inflammatory pathways. Compared with RDFP-U, RDFP-UAE demonstrated greater inhibitory activity under the experimental conditions evaluated, highlighting its potential for further investigation as a functional ingredient in inflammation-related contexts.

### 3.4. Effects of RDFPs on proinflammatory cytokine production

**Figure 5:**
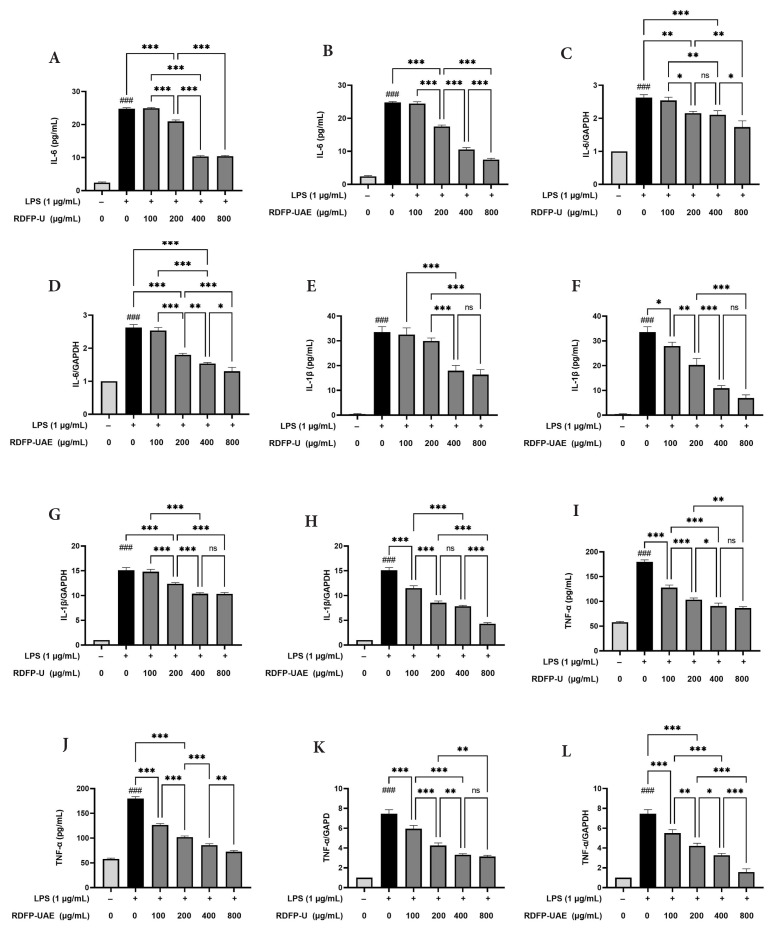
Effects of RDFP-U and RDFP-UAE extracted from red dragon fruit on proinflammatory cytokines (IL-6, IL-1β, and TNF-α) in RAW 264.7 macrophages. (A–D) Protein levels and mRNA expression of IL-6. (E–H) Protein levels and mRNA expression of IL-1β. (I–L) Protein levels and mRNA expression of TNF-α. Data are presented as mean ± SEM (n = 3). ns, not significant; ### p < 0.001 versus control; * p < 0.05; ** p < 0.01; *** p < 0.001 versus LPS.

To better understand the impact of RDFP-U and RDFP-UAE on inflammatory signaling, the levels of proinflammatory cytokines (IL-6, IL-1β, and TNF-α) were assessed using ELISA, and their mRNA expression was analyzed via RT-qPCR in LPS-stimulated RAW 264.7 cells (Figure 5). Compared with the control group, LPS stimulation significantly increased IL-6, IL-1β, and TNF-α levels (p < 0.01), reaching 24.79 pg/mL, 33.55 pg/mL, and 178.75 ng/mL. Pretreatment with RDFP-U and RDFP-UAE significantly inhibited LPS-induced secretion of IL-6 and IL-1β (p < 0.01). Similarly, TNF-α secretion was markedly suppressed, with RDFP-UAE showing the strongest inhibitory effect (p < 0.01).

At the mRNA level, LPS stimulation significantly upregulated IL-6, IL-1β, and TNF-α expression in RAW 264.7 cells. Treatment with RDFP-U and RDFP-UAE reduced these expression levels in a dose-dependent manner, with the greatest inhibition observed at 800 μg/mL. These findings suggest that RDFP-U and RDFP-UAE suppress the production of proinflammatory cytokines in activated macrophages under the conditions tested.

LPS, a potent endotoxin, activates macrophages via TLR4 signaling, triggering NF-κB and mitogen-activated protein kinase pathways that upregulate proinflammatory cytokines. IL-6, IL-1β, and TNF-α amplify immune responses, contributing to tissue damage and chronic inflammation in disorders such as IBD and rheumatoid arthritis (Cavaillon, 2018). In this study, RDFP-U and RDFP-UAE significantly inhibited both cytokine secretion and mRNA expression, with RDFP-UAE demonstrating greater and more consistent inhibitory activity under the experimental conditions evaluated. This suggests that ultrasound-assisted enzymatic extraction may enhance the antiinflammatory properties of pectin, potentially through alterations in structural characteristics, such as changes in molecular weight distribution and galacturonic acid content, which could influence its interaction with immune cells.

The present findings are consistent with previous reports describing the ability of pectins to downregulate proinflammatory cytokines. For example, highly purified low-molecular-weight citrus pectins (USP1, USP2, and USP3) have been reported to inhibit NO production, reactive oxygen species (ROS) generation, and the production of proinflammatory cytokines (TNF-α, IL-6, and IL-1β). The monosaccharide composition of pectins is closely linked to their immunomodulatory activity. Similar trends were reported by Wang et al. (2025), who found that galactose and glucuronic acid in ASPS-A1 from *Acanthopanax senticosus* were associated with cytokine modulation in RAW 264.7 macrophages, and by Zheng et al. (2025), who observed that XGJ-5 polysaccharides with high glucuronic acid content (63.4%) from longan pulp modulated TNF-α and IL-6 levels. Previous studies support this association, suggesting that pectins with lower molecular weight and higher galacturonic acid or specific neutral sugar content may exhibit enhanced immunomodulatory effects. For example, purified low-molecular-weight citrus pectins and polysaccharides rich in galactose and glucuronic acid have been reported to reduce NO and ROS production, as well as proinflammatory cytokine expression, in macrophages.

## 4. Conclusion

In summary, this study demonstrates that pectin extracted from red dragon fruit peel using ultrasound-assisted enzymatic extraction (RDFP-UAE) exhibited greater antiinflammatory activity than pectin obtained through ultrasound-assisted extraction alone (RDFP-U) under the experimental conditions alone. RDFP-UAE exhibited a lower degree of esterification and molecular weight, but a significantly higher galacturonic acid content, compared with RDFP-U. Both extracts were noncytotoxic and significantly reduced key inflammatory mediators, including nitric oxide production, iNOS and COX-2 expression, PGE_2_ levels, and proinflammatory cytokine production (IL-6, IL-1β, and TNF-α) in LPS-stimulated RAW 264.7 macrophages. Notably, RDFP-UAE consistently showed greater inhibitory effects, which may be associated with extraction-induced structural differences, including variations in molecular weight distribution and galacturonic acid content. These structural differences may influence the biological activity of pectin in macrophages; however, the precise molecular mechanisms require further investigation.

These findings highlight the importance of extraction techniques in optimizing the functional properties of pectin, and further investigations, including in vivo safety and efficacy studies, are warranted.

## Figures and Tables

**Table:** Polysaccharide yield, chemical composition, and molecular weight of RDFP-U and RDFP-UAE.

Polysaccharide		RDFP-U	RDFP-UAE	p
Yield (g/100 g)		12.63 ± 0.28	19.38 ± 0.36	<0.0001
Degree of esterification (%)		75.82 ± 1.84	46.51 ± 2.41	<0.0001
Molecular weight (kDa)		178.25 ± 3.17	94.67 ± 5.41	<0.0001
Monosaccharide composition (%, w/w)	Galactose	10.31 ± 2.74	6.32 ± 0.11	0.0652
	Galacturonic acid	38.74 ± 0.45	67.51 ± 4.39	0.0003
	Mannose	11.04 ± 1.54	4.64 ± 0.78	0.0028
	Rhamnose	14.32 ± 4.11	10.87 ± 1.19	0.2351
